# Noninvasive treatment for chronic sialadenitis: Case report

**DOI:** 10.1002/ccr3.2379

**Published:** 2019-08-20

**Authors:** Farnoosh Razmara, Xaniar Mahmoudi

**Affiliations:** ^1^ Department of Oral and Maxillofacial Surgery Tehran University of Medical Sciences Tehran Iran; ^2^ School of Dentistry, International Campus, Department of Oral and Maxillofacial Surgery Tehran University of Medical Sciences Tehran Iran

**Keywords:** noninvasive treatment, salivary gland disease, sialadenitis, submandibular gland

## Abstract

This reporter expresses a patient with a history of tender neck swelling. After clinical and graphical examinations, the patient was diagnosed with submandibular sialolithiasis. Instead of invasive removal of the gland, a more conservative treatment was used. Eventually, after a 1‐year‐long follow‐up, the patient's conditions were found to be acceptable.

## 1 INTRODUCTION

Chorionic sialadenitis is an inflammatory condition in which a patient suffers from periodic swelling and pain in the salivary gland due to insufficient saliva secretion. Progressive bacterial infection is expected to occur due to reduced salivary flow. Recurrence of these infections leads to the formation of sialectasis, acinar destruction, fibrosis, and infiltration of chronic inflammatory cells, which further reduces the flow of saliva.^1^, ^2^ Sialolith is the main cause of obstructive sialadenitis.

Sialolithiasis mostly occurs in the major salivary glands and refers to the presence of calcified structures within the duct of major or minor salivary glands.^3^

The exact etiology of sialolith is unknown, but some researchers believe that sialolith is the result of salivary stasis and spasmodic contractions of the ducts due to factors such as saliva properties, chemical changes, salivary accumulation, foreign bodies, bacterial infections, and inflammation.^4^ Features such as longer length of the major duct and consistency and characteristics of the saliva secreted by the submandibular gland have caused most of the obstructions appear in this gland.^5^ Most cases (80%‐90%) occur in the submandibular gland, followed by parotid (5%‐10%) and sublingual (<1%) glands.^6^ About 40% of the submandibular calculi are located in the distal part of the duct and can be removed by surgical procedures under local anesthesia. Sialoadenectomy is the treatment of choice for calculi in the proximal part of the duct or within the submandibular gland.^7^

Conservative treatment is usually used to treat cases with sialadenitis and surgical procedures such as papillotomy and sialoadenectomy are reserved for refractory patients.^8^

The aim of this study was to report a case of submandibular sialolithiasis treated with a conservative approach, including removing the calculi and suturing the duct to the floor of the mouth.

## 2 CASE REPORT

**Figure 1:**
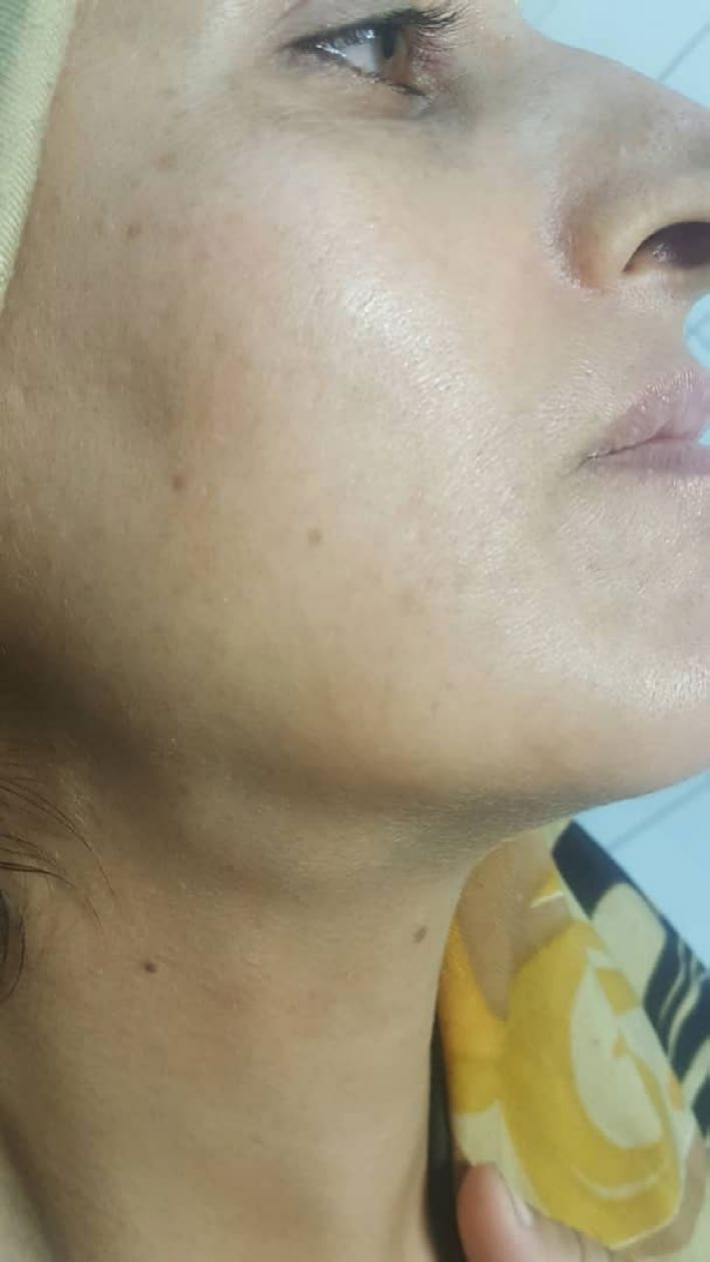
Extraoral swelling is seen in clinical examination

**Figure 2:**
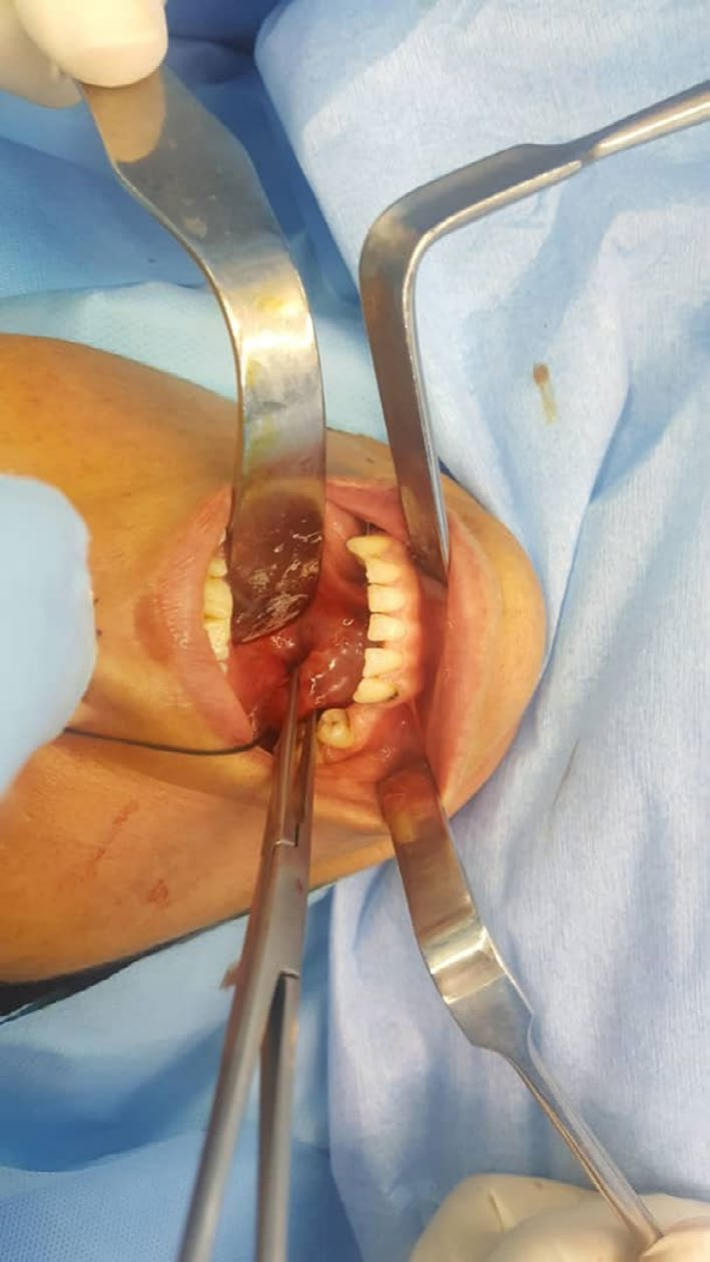
Intraoral view of the submandibular stone

**Figure 3:**
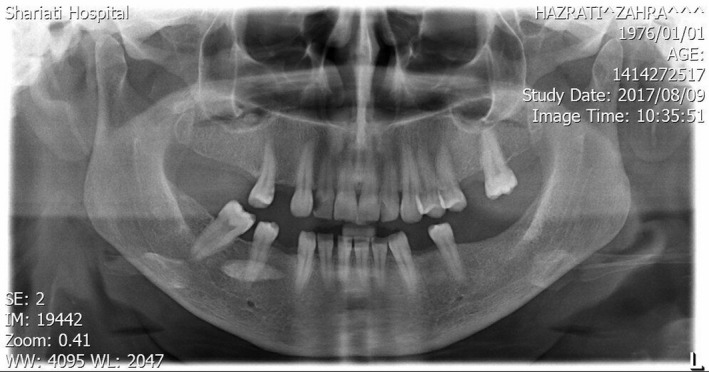
Preoperative panoramic view, a radiopaque body appears in the right submandibular gland

**Figure 4:**
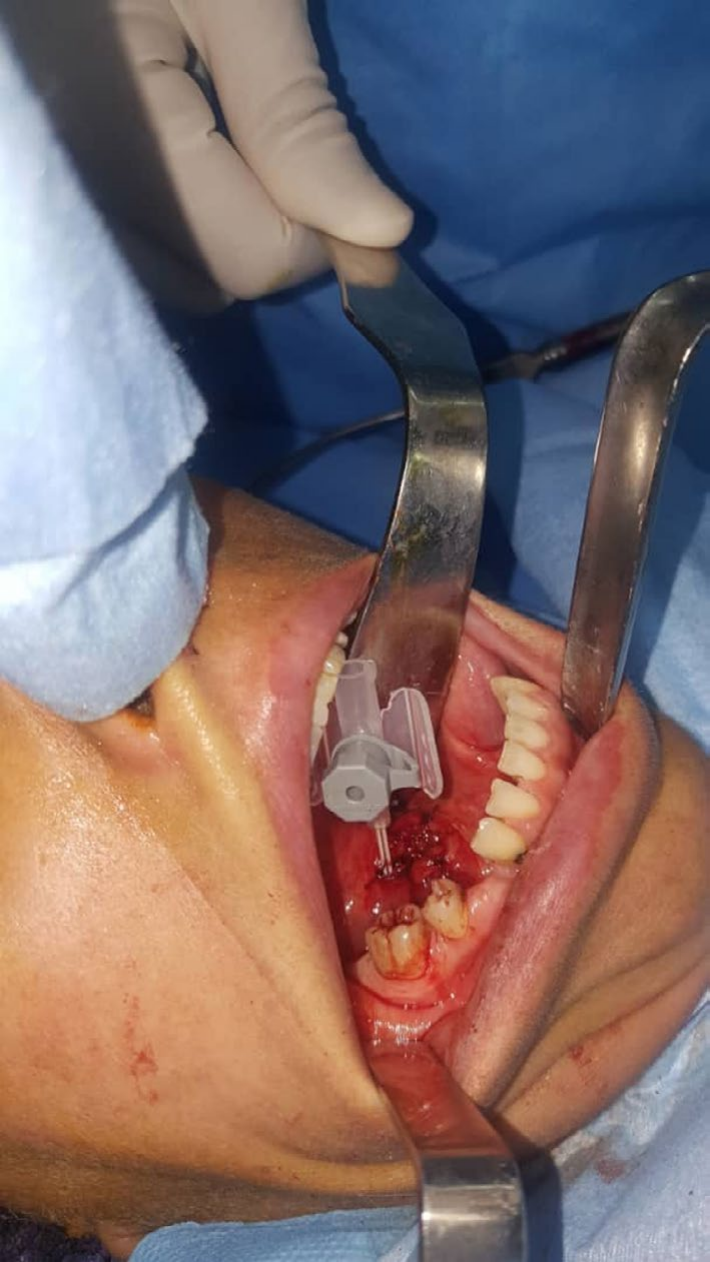
Wharton's duct cannulation

**Figure 5:**
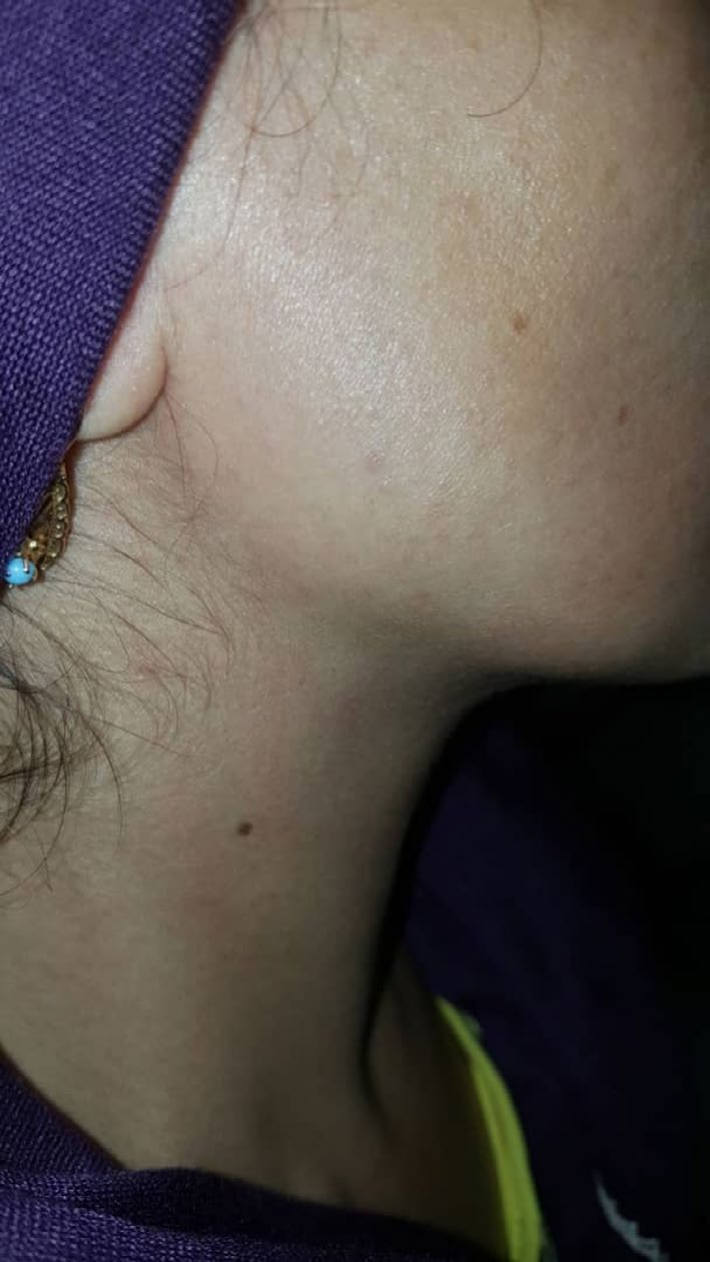
Patients swelling is cured after 12 mo

**Figure 6:**
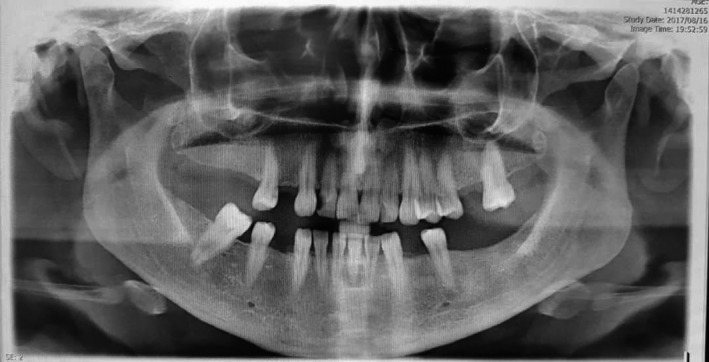
Postoperative panoramic view

A 37‐year‐old woman referred to our clinic with complaints of neck swelling since the past 2 years, which was tender and had infectious secretions after a few months. This problem was first seen only during eating and got slightly better with traditional herbal remedies. After a year, previous treatments did not work, and the patient was annoyed by a permanent pain. In clinical examinations, a solid swelling was observed in the right lateral region of the neck under the jaw angle, and regional lymphadenopathy was evident (Figure 1). Suppuration secretion was not clearly visible in the mouth. A stiff and painful swelling was seen in the mouth near the Wharton duct (Figure 2). After taking panoramic view, sialadenitis was diagnosed to have occurred due to sialolith in the right submandibular gland (Figure 3). Regarding the chronicity of sialadenitis and the history of infectious secretions in the submandibular gland, the selective treatment was removal of the gland by surgery. However, to prevent possible injuries such as damage to the marginal mandibular nerve and skin scar after healing, it was preferred to use a more conservative treatment. The risk of recurrence of the lesion was explained to the patient. Due to the presence of stones above the mylohyoid muscle, it was decided to remove it by intraoral approach. After initial medical evaluations, the patient was placed under general anesthesia and stone was removed through a longitudinal cut along the duct of the salivary glands. Our goal was to cannulation the salivary gland duct, which was done using a pink peripheral venous catheter with an external diameter of 1/1 and an internal diameter of 0/8 (Figure 4). First, the catheter was placed into the Wharton duct by a needle. Then, the needle was removed and the catheter remained in the duct. Several back and forth movements were performed in the direction of the duct to remove the obstruction, and the orifice of Wharton was sutured to the floor of the mouth. Narrowing the Wharton orifice would prevent the saliva from escaping, and the obstruction would not come back in the near future. The patient's conditions were satisfactory after a 1‐year follow‐up (Figure 5), and no opacity was seen in the panoramic view (Figure 6).

## 3 DISCUSSION

Sialadenitis is the most common disease that affects the salivary glands and is the most common reason for submandibulectomy. In addition to gland resection, the remaining ducts should be cleared from stone. Otherwise, it can cause abscesses in the area of the calculus even several years later. This indicates the importance of complete purification of the gland and associated ducts. General anesthesia is required for submandibulectomy, and nerve weakness and scar formation are possible to occur.^9^

Clinical examinations along with panoramic and occlusal views can assist the clinicians in the process of diagnosis. However, some researchers have recommended the application of more imaging techniques such as ultrasound, computed tomography, CBCT, and contrast sialography. The signs and symptoms of salivary flow obstruction are well defined, which include formation of transient local edema and pain before and during meals and progressive postprandial remission. Further, chronic recurrent duct obliteration is able to bring about inflammation and infection.^3^, ^10^

In cases with small stones, conservative management can be used along with local heat, massage, and sialogogues. Infections should be treated with antibiotics together with simple sialolithotomy if needed.^11^, ^12^ If the stone is located in the distal one‐third of the duct, a simple surgical release can be carried out by making an incision on the mouth floor. Making an incision in the duct transorally can remove more posterior stones. Care should be taken since the lingual nerve is located posteriorly to the submandibular duct, and the two structures are tightly related. If the gland is impaired by recurrent infections and fibrosis, or calculi are developed in the gland, removal of the gland may be required.

Salivary gland resection, which is associated with risk of nerve damage, esthetic problems, and longer hospitalization, has been reported to decrease and require minimally invasive therapies. Several types of minimally invasive treatments, including interventional sialendoscopy (iSGE), extracorporeal shock wave lithotripsy (ESWL), and combined endoscopic surgical procedures, have been proposed for the treatment of salivary gland stones.^13^ Choosing the right treatment should involve criteria such as the involved gland, the number and size of stones, location, and the relation to the duct. A method with the least damage is always the treatment of choice.^14^ Postoperative complications such as pain, scar, and nerve damages often happen. Disorders such as xerostomia (up to 31%), alteration of taste (16.3%), hematomas (up to 14%), facial nerve damage (8%), and lingual nerve damage (12%) have been observed in the treated patients.^15^, ^16^, ^17^, ^18^, ^19^ Sialoendoscopy is used to eliminate and treat the obstructions and decrease the probable injuries. As a minimally invasive technique, sialoendoscopy is used for the treatment of the ductal system obstructions. If the stones are large or shaped irregularly, it can be utilized in combination with surgical and other fragmentation procedures (eg, laser and shock wave lithotripsy).^20^

Many cases have been surgically treated with local anesthetic successfully. Although general anesthesia allows practitioners to better control patients in long‐term surgeries in harder cases,

Functional recovery of the glands and absence of inflammation, fibrosis, and atrophy have been observed in histopathological images.^21^

## 4 CONCLUSION

Wharton's duct sialolith is one of the most common causes of submandibular sialadenitis. If the stone is removed promptly from the duct, it prevents the development of sialadenitis, infectious secretions, and regional lymphadenopathy, and glandular parenchyma does not disappear ultimately. In cases where chronic sialadenitis occurs due to the presence of stones in the area, it is advisable to initiate conservative treatments on the agenda. If the patient's conditions are not improved, more aggressive treatments should be considered. One should not forget the patient's annual follow‐up.

## CONFLICT OF INTEREST

None declared.

## AUTHOR CONTRIBUTION

FR and XM: was involved in the conception and design of the work, data collection, drafting of the manuscript, critical revision of the manuscript, and final approval of the version to be published.
